# Satisfaction with life and character strengths of non-religious and religious people: it’s practicing one’s religion that makes the difference

**DOI:** 10.3389/fpsyg.2014.00876

**Published:** 2014-08-14

**Authors:** Anne Berthold, Willibald Ruch

**Affiliations:** Department of Psychology – Personality and Assessment, University of ZurichZurich, Switzerland

**Keywords:** character strengths, religiousness, satisfaction with life, orientations to happiness, practicing religion

## Abstract

According to systematic reviews, religious beliefs and practices are related to higher life satisfaction, happiness, and positive affect (Koenig and Larson, 2001). The present research extends previous findings by comparing satisfaction with life and character strengths of non-religious people, religious people, who practice their religion and people that have a religious affiliation but do not practice their religion. We assessed life satisfaction (SWLS), character strengths (VIA-IS) and the orientations to happiness (OTH) in a sample of *N* = 20538 participants. People with a religious affiliation that also practice their religion were found to be more satisfied with their life and scored higher on life of meaning than those who do not practice their religion and than non-religious people. Also religious people who practice their religion differed significantly from those who do not practice their religion and non-religious people regarding several character strengths; they scored higher on kindness, love, gratitude, hope, forgiveness, and on spirituality. There were no substantial differences between people who had no religious affiliation and those with a religious affiliation that do not practice their religion (all $\eta_{p}^{2}$*s* < 0.009). Altogether, the present findings suggest that people profit from a religious affiliation if they also actively practice their religion.

## INTRODUCTION

### SATISFACTION WITH LIFE AND RELIGION

One pertinent research question in Positive Psychology is the identification of variables that enable a fulfilling, meaningful, and happy life. In the past years a sizable amount of studies has been accumulated to examine the relation of character strengths and well-being (e.g., Isaacowitz et al., 2003; Park et al., 2004). It was consistently found that life satisfaction is particularly related to the five following scales of the *Values in Action Inventory of Strengths* (VIA-IS; Peterson and Park, 2004; Peterson et al., 2005a): *curiosity, zest, love, gratitude,* and *hope*.

Reviewing previous studies using the VIA-IS, we found no consistent pattern regarding the relation of life satisfaction and the strength religiousness, which is defined as having coherent beliefs about the higher purpose and meaning of the universe, knowing where one fits within the larger scheme. Correlation coefficients vary from significantly negative (e.g., *r* = -0.14; Karris, 2009) to positive (e.g., *r* = 0.32; Peterson et al., 2007). Yet, the inconsistency of these results is not surprising. Numerous studies already examined the relation of religiousness and mental health, psychological distress, and other variables related to well-being using a variety of measures for the assessment of religiousness (see Saroglou, 2014, for a review). On the one hand, researchers concluded that the relation is weak and negative (Batson et al., 1993; Ellison and Lee, 2010), on the other hand, systematic meta-analyses point in the other direction (Koenig and Larson, 2001; Smith et al., 2003). The aim of the present research is to identify a condition under which a positive influence of religiousness on life satisfaction can be expected.

One reason for the discrepancy among previous studies (using the VIA-IS) might be the variation of unnoticed, but important, third variables (e.g., a social norm or the exertion of religious behaviors). Recently, Stavrova et al. (2013) showed that religious people are happier and more satisfied with life than non-religious individuals especially in countries with a positive social norm toward religiousness (see also Gebauer et al., 2012). The influence of such a social norm could be multifaceted. Aside from feeling socially supported, the perceived fit between religious values and environment could encourage religious people to engage in practicing their religion more actively, which in turn could lead to more satisfaction with life.

It is assumed that the use of one’s strengths is fulfilling and that well-being is enhanced via the development of one’s signature strengths (i.e., participants top five strengths). A number of studies applying strengths-intervention programs provide evidence for an increase in well-being after the strengths-based intervention (Seligman et al., 2005; Rust et al., 2009; Proctor et al., 2011; Gander et al., 2013; Rashid, in press). The findings of Harzer and Ruch (2013) also support this reasoning by showing that job satisfaction increased along with increasing numbers of signature strengths applicable at the workplace. Aside from research using the VIA-IS, Sagiv and Schwartz (2000) provide evidence that well-being is fostered when there is congruence between individuals’ values and their environment. They argue: “people are more likely to experience positive well-being when they can express and fulfill their values …” (p. 186). According to this theorizing – and in line with previous results – religious individuals should be more satisfied with their lives when they also practice their religion.

But, are prayers really necessary? Sethi and Seligman (1993) analyzed nine different religions and found a positive relation between religiousness and hope as well as optimism. Their analyses revealed that the liturgy (i.e., religious material) plays an important role. Other relevant factors positively related to optimism were religious influence in daily life, religious involvement and religious hope (see also Ciarrocchi et al., 2008). Further evidence for the influence of prayers can be found in a study of Mochon et al. (2008) focusing on the frequency of religious practice. The authors suggest that regular activities (e.g., attending religious services) can provide people with small positive boosts. The findings showed that well-being was more positive, the more often religion was practiced. However, the same authors also concluded that the relationship between religiousness and well-being is more complex than they had thought (Mochon et al., 2011). They found that people who claim to be religious only to a small extent are less happy than non-believers.

Additional support for our hypothesis that a religious affiliation alone does not automatically lead to higher life satisfaction is provided by Salsman et al. (2005). They showed that extrinsic religiousness had no influence on satisfaction with life, while intrinsic religiousness and prayer fulfillment was positively related to life satisfaction. Also, Lambert et al. (2009) showed in a set of studies that praying could increase gratitude (one of the strengths that is strongly related to life satisfaction).

Summing up – and in accordance with previous results – we hypothesized that satisfaction with life is increased in religious people who practice their religion compared to religiously affiliated people that do not practice their religion. This latter group was expected to be as satisfied with their lives as people without a religious affiliation.

### CHARACTER STRENGTHS AND RELIGION

The inspection of historical texts across different cultures and religions enabled researchers to identify six ubiquitous virtues (Dahlsgaard et al., 2005). Later, 24 character strengths were derived that enable the virtues: creativity, curiosity, open-mindedness, love of learning, perspective, authenticity, bravery, perseverance, zest, kindness, love, social intelligence, fairness, leadership, teamwork, forgiveness, humility, prudence, self regulation, appreciation of beauty and excellence, gratitude, hope, humor, and religiousness (Peterson and Seligman, 2004).

Studying religions had been particularly important for the creation of the list of character strengths. However, studies that directly investigate the influence of being religious or practicing religion on character strengths are fairly scarce in positive psychology research. Moreover, those few studies comparing religious and non-religious individuals that use the VIA-IS yielded contradictory results. Bai (2011, Unpublished Doctoral dissertation) for example found no differences between individuals with and without a religious denomination. Ahmed (2009), on the other hand, showed that various character strengths depended on the individual’s religiousness. The inconsistency of previous results calls for more research to shed light on the manifold influences of religion on character strengths. The present research aims to contribute to the understanding of this relationship.

There are several possibilities available to generate hypotheses regarding religiousness and character strengths. Reviewing religious readings is a helpful alternative to collect exploratory hypotheses and it becomes apparent that religious norms are related to quite a few character strengths, as for example *gratitude, kindness,* and *forgiveness*. The rules of gratitude, kindness and forgiving are omnipresent lessons taught through religious readings and services. A relationship between these strengths and religiousness has also already been reported in previous research. There is for example empirical evidence that praying increases gratitude (Lambert et al., 2009). Thus, the character strength *gratitude* should be particularly increased in religious people that practice their religion. The strength gratitude was also included in research of Ahmed (2009), who showed that highly religious participants (i.e., American Muslim youth) were characterized by the strengths: kindness, equity, leadership, self-regulation, prudence, gratitude, hope/optimism, spirituality, and forgiveness. We therefore assume that these strengths are increased in religious people particularly when they practice their religion.

Another aid in the generation of hypotheses regarding character strengths is to examine the literature on values and religiousness. A meta-analysis based on data of the Schwartz value survey revealed that religious people favor conservative values such as tradition, conformity and tend to dislike hedonism (Saroglou et al., 2004). However, a differential investigation of the facets of religiousness showed a more complex picture. Saroglou and Munoz-Garcia (2008) showed that the preference for different values varied depending on which facet of religiousness was being considered. Religiosity was positively associated with conformity, while spirituality and conformity were unrelated. All religiousness measures, however, were positively related to the value of benevolence. The value benevolence, in a way, corresponds to the strengths forgiveness and kindness. Thus, the findings regarding values and religiousness support our assumption that peoples’ character strengths (especially *kindness* and *forgiveness*) vary depending on their religiousness.

### ORIENTATIONS TO HAPPINESS AND RELIGION

According to Seligman (2002), people can take three different roads to achieve happiness. The pleasurable route to happiness is characterized by a *hedonistic* worldview – accentuating the quest for pleasure and avoidance of pain (Peterson et al., 2005b). The second orientation to happiness is the life of engagement, which is based on Csikszentmihalyi’s (1990) concept of flow. Through engaging in an activity, people can become absorbed, and receive gratification from these actions. In addition to pleasure and engagement, there is a third route to happiness: the life of meaning. This third route emphasizes that people can obtain happiness by identifying one’s virtues and by living in accordance with this virtues to accomplish a higher purpose in life.

Previous research on the three orientations to happiness (OTH) showed that religious people differ regarding the two orientations pleasure and meaning (Ruch et al., 2010a). Life of meaning was found to be higher in religious people, while, in contrast, pleasure appears to be negatively related to religiousness (see also Peterson et al., 2007). In line with previous findings, we expected that religious individuals would score high on meaning and low on pleasure. Support for the latter hypothesis can be partly derived from research on values and religion, showing that hedonism is less appreciated in religious people (Saroglou et al., 2004). However, research addressing religion and the OTH in more detail is lacking. Thus, we analyzed pleasure, engagement and meaning of religious people – in particular of those who practice their religion.

Altogether, the present research examines the relation of religiousness and the variables that define “the good life” – character strengths, satisfaction with life and the OTH. Comparing non-religious individuals with religiously affiliated individuals who either do practice vs. do not practice their religion, it was predicted that individuals who indicated that they practiced their religion would score higher on life satisfaction and would also differ substantially from the other respondents regarding character strengths and OTH (especially life of meaning).

## MATERIALS AND METHODS

### PARTICIPANTS

The sample consisted of 20538 German-speaking respondents. About 56% of the participants were from Germany, 31% were Swiss and 10% were from Austria. The respondents had a mean age of 39 years (SD = 12.39) with a range across the adult years. There were more female (70%, *n* = 14375) than male participants. About 48% (*n* = 9848) participants of our sample were married or in a relationship, <40% (*n* = 7999) were single, 12% (*n* = 2442) were separated or divorced, and ca. 1% (*n* = 249) were widowed. One third of our participants (*n* = 6168) were living alone, more than 50% (*n* = 10472) were living together with their partner or marriage partner, 9.4% (*n* = 1938) were living in a flat-sharing community, and 9.5% (*n* = 1960) were living together with their parents.

Almost 80% (*n* = 16210) of our participants reported themselves to be employed. About 57% (*n* = 11625) had a university degree, 20% (*n* = 4154) reported the completion of primary, required education, 13% (*n* = 2606) had a baccalaureate, 10% (*n* = 2077) of them had completed an apprenticeship, and <1% (*n* = 76) left school after the primary level.

### INSTRUMENTS

#### Religious affiliation and practice of religion

One single item was used to assess participants’ religious denominations. 25.5% of the participants indicated themselves to have no religious affiliations (*n* = 5235), the other 74.5% (*n* = 15303) were religiously affiliated. Next, we assessed whether participants practiced their religion or not with one item: “Do you practice your religion?” (1 = yes, 2 = no, 3 = no religion). 30% of the participants indicated to practice their religion (*n* = 6165) and 44.5% did not practice their religion (*n* = 9138).

Twenty-six percent of the female and 24% of the male participants were not religiously affiliated. Approximately 31% of the female sample and 28% of the male participants indicated to practice their religion and 43% of the female participants vs. 48% of the males had a religious affiliation but did not practice their religion. According to Cramérs *V* (φ_c_ = 0_._04), there appears to be no meaningful relationship between gender and religious participation.

#### Values in action inventory of strength (VIA-IS)

The Values in Action Inventory of Strengths (VIA-IS; Peterson et al., 2005a) consists of 240 items for the self-assessment of the 24 character strengths (10 items per strength) included in the classification of Peterson and Seligman (2004). The questionnaire uses a 5-point Likert-scale (*very much like me* to *very much unlike me*). Example items are *“I believe in a universal power, a god.”* (*religiousness*), *“I have voluntarily helped a neighbor in the last month.” (kindness), “I never miss group meetings or team practices.” (teamwork), “I try to respond with understanding when someone treats me badly.” (forgiveness).* We used the German translation (Ruch et al., 2010b) of the VIA-IS that has comparable psychometric properties to the US-version. It has been used in numerous studies (e.g., Müller and Ruch, 2011; Proyer et al., 2011; Güsewell and Ruch, 2012). In the present sample the alpha coefficients were between α = 0.71 (*authenticity*) and α = 0.90 (*religiousness*) with a median of α = 0.78.

#### Orientations to happiness scale

The OTH Scale (Peterson et al., 2005b) is a self-report questionnaire for the assessment of the three OTH (Life of Pleasure, Life of Engagement, Life of Meaning). The questionnaire uses a 5-point Likert-scale (*very much unlike me* to *very much like me*) and consists of 18 items (each scale is measured with six items). A sample item for the assessment of life of engagement is *“Regardless of what I am doing, time passes very quickly.”* We used the German version of the OTH (Ruch et al., 2010b) that has shown good psychometric properties in several studies (e.g., Proyer et al., 2012). In the present sample the alpha coefficients of the three scales of the OTH were α = 0.74* (pleasure),* α = 0.65* (engagement)*, and α = 0.78* (meaning)*.

#### Satisfaction with life scale

The Satisfaction with Life Scale (SWLS; Pavot and Diener, 2008) is a 5-item measure that is widely used in research for the assessment of life satisfaction (as a global cognitive judgment of one’s own life). The scale showed good psychometric properties across several studies (e.g., Diener et al., 2000). We used the German translation of the scale that has shown equally good psychometric properties (e.g., Peterson et al., 2007; Ruch et al., 2010b). A sample item is *“The conditions of my life are excellent.”* It uses a 7-point answer format (ranging from *strongly disagree* to *strongly agree*). The SWLS had a high internal consistency in our sample (α = 0.87).

### PROCEDURE

The respondents completed all three questionnaires (VIA-IS, OTH, SWLS) via the German online platform: www.charakterstaerken.org. They registered, provided socio-demographic information, and completed the measures sections. None of the participants was paid. Immediately after completion, all participants received individualized feedback on their results. Though data collection via the Internet is sometimes criticized, there are empirical studies showing that Internet-based studies are usually as reliable and valid as more traditional strategies (e.g., paper–pencil) and that web-based samples are usually more diverse than other samples (Gosling et al., 2004; Howell et al., 2010). All local ethical guidelines were fulfilled.

## RESULTS

Given the size of our sample, any two means that differed by 0.02 or more were statistically different (*p* < 0.05). Thus, we decided to consider and refer to the effect sizes (i.e., $\eta_{p}^{2}$) when reporting effects. By applying this strategy, we aimed to reduce the risk of overestimating the differences between our comparison groups (i.e., non-religious individuals, religiously affiliated individuals who practice their religion and not practice their religion). Effect sizes $\eta_{p}^{2}$*s* of ≥0.01 were considered as indicative.

### RELIGIOUSNESS, PRACTICING RELIGION, AND SATISFACTION WITH LIFE

To compare the life satisfaction of non-religious participants, religiously affiliated participants, who practice their religion and those who not practice their religion, we conducted a univariate ANOVA (analysis of variance) with the dependent variable “satisfaction with life” and the independent factor group (religious and practicing, religious and not practicing, non-religious individuals). The means and standard deviations are reported in **Table 1**. The effect sizes indicated that, as predicted, religious people, who practice their religion, reported a higher satisfaction with life than their counterparts, who did not practice their religion and also a higher life satisfaction than non-religious individuals. There was no substantial difference between non-religious individuals and religiously affiliated participants, who did not practice their religions (see **Table 2** for *F*-values and effect sizes).

**Table 1:** Reliabilities of the VIA-IS-scales, SWLS, OTH, and descriptive statistics of the three groups.

		Religiously affiliated and practicing individuals		Religiously affiliated and not practicing individuals		Non-religious individuals	
	α	*M*	SD	*M*	SD	*M*	SD
Creativity	0.88	3.58	0.65	3.51	0.66	3.60	0.65
Curiosity	0.81	4.03	0.51	3.91	0.56	3.95	0.55
Open-mindedness	0.80	3.86	0.47	3.84	0.49	3.89	0.48
Love of learning	0.83	3.92	0.57	3.82	0.60	3.92	0.58
Perspective	0.77	3.59	0.48	3.55	0.50	3.56	0.49
Bravery	0.77	3.61	0.53	3.52	0.55	3.60	0.54
Persistence	0.86	3.53	0.60	3.41	0.64	3.42	0.65
Honesty	0.71	3.84	0.44	3.79	0.44	3.79	0.44
Zest	0.80	3.66	0.56	3.55	0.59	3.53	0.60
Love	0.78	3.92	0.51	3.77	0.57	3.74	0.58
Kindness	0.73	3.91	0.47	3.80	0.48	3.78	0.48
Social intelligence	0.76	3.74	0.46	3.69	0.49	3.68	0.50
Teamwork	0.75	3.68	0.47	3.58	0.48	3.52	0.50
Fairness	0.76	3.95	0.46	3.89	0.48	3.86	0.47
Leadership	0.76	3.68	0.48	3.59	0.48	3.56	0.49
Forgiveness	0.80	3.61	0.54	3.48	0.54	3.47	0.56
Modesty	0.78	3.29	0.58	3.21	0.57	3.14	0.57
Prudence	0.73	3.40	0.53	3.32	0.54	3.31	0.54
Self-regulation	0.74	3.26	0.56	3.14	0.58	3.16	0.58
Appreciation of beauty	0.75	3.72	0.53	3.57	0.56	3.60	0.57
Gratitude	0.81	3.91	0.51	3.62	0.53	3.62	0.56
Hope	0.83	3.64	0.59	3.49	0.64	3.46	0.65
Humor	0.86	3.66	0.61	3.65	0.61	3.63	0.61
Religiousness	0.90	3.78	0.66	2.61	0.75	2.51	0.82
SWLS	0.87	4.86	1.20	4.65	1.26	4.55	1.31
OTH – pleasure	0.74	3.23	0.71	3.38	0.70	3.38	0.70
OTH – engagement	0.65	3.16	0.63	3.08	0.64	3.07	0.65
OTH – meaning	0.78	3.42	0.77	2.96	0.79	2.90	0.83

*α = Cronbach’s α, N = 20538, religiously affiliated and practicing individuals n = 6165, religiously affiliated and not practicing individuals n = 9138, non-religious individuals n = 5235. SWLS, Satisfaction with Life; OTH, Orientation to Happiness; M, Mean, SD, Standard Deviation.*

**Table 2:** *F* -values for the comparison of the three groups regarding character strengths, SWLS, and OTH.

	Comparison of individuals with religious affiliation that *practice* (=1) vs. *not practice* (=2) their religion		Comparison of individuals with religious affiliation that *practice* their religion vs. *non-religious* (=3) individuals		Comparison of individuals with religious affiliation that do *not practice* their religion vs. *non-religious* individuals	
	*F*	$\eta_{p}^{2}$	*F*	$\eta_{p}^{2}$	*F*	$\eta_{p}^{2}$
Creativity	32.47	0.00	2.64	0.00	51.32	0.00
Curiosity^a,b^	204.10	0.01	69.78	0.01	20.44	0.00
Open-mindedness	8.54	0.00	10.76	0.00	38.74	0.00
Love of learning^a,c^	119.14	0.01	0.03	0.00	110.54	0.01
Perspective	28.39	0.00	12.28	0.00	1.68	0.00
Bravery^a^	102.43	0.01	1.28	0.00	70.10	0.00
Persistence^a,b^	124.20	0.01	92.72	0.01	0.02	0.00
Honesty	49.82	0.00	39.59	0.00	0.01	0.00
Zest^a,b^	146.66	0.01	157.75	0.01	3.92	0.00
Love^a,b^	279.07	0.02	308.09	0.03	8.55	0.00
Kindness^a,b^	179.43	0.01	200.21	0.02	6.91	0.00
Social intelligence	39.42	0.00	39.72	0.00	0.66	0.00
Teamwork^a,b^	156.94	0.01	323.37	0.03	59.39	0.00
Fairness^b^	58.67	0.00	100.13	0.01	11.72	0.00
Leadership^a,b^	132.24	0.01	177.24	0.02	12.91	0.00
Forgiveness^a,b^	223.60	0.01	196.42	0.02	1.41	0.00
Modesty^a,b^	79.42	0.01	190.66	0.02	43.02	0.00
Prudence^a,b^	91.14	0.01	77.89	0.01	0.23	0.00
Self-regulation^a,b^	150.07	0.01	93.72	0.01	1.37	0.00
Appreciation of Beauty^a,b^	265.58	0.02	133.89	0.01	8.26	0.00
Gratitude^a,b^	1128.64	0.07	879.29	0.07	0.62	0.00
Hope^a,b^	218.84	0.01	239.93	0.02	6.68	0.00
Humor	0.37	0.00	4.49	0.00	2.93	0.00
Religiousness^a,b^	9815.68	0.39	8413.89	0.42	58.03	0.00
SWLS^a,b^	110.96	0.01	174.90	0.02	18.65	0.00
OTH – pleasure^a,b^	168.21	0.01	130.57	0.01	0.01	0.00
OTH – engagement	61.80	0.00	56.03	0.00	0.43	0.00
OTH – meaning^a,b^	1276.90	0.08	1197.96	0.10	17.96	0.00

*N = 20538, SWLS = Satisfaction with Life, OTH = Orientation to Happiness, religious affiliation (1 = yes; 2 = yes, but not practicing, 3 = no), F = F-value, $\eta_{p}^{2}$ = effect size eta square.*

*^a^Substantial differences between religiously affiliated individuals that practice vs. not practice their religion.*

*^b^Substantial differences between religiously affiliated individuals that practice their religion and non-religious individuals.*

*^c^Substantial differences between religiously affiliated individuals that do not practice their religion and non-religious individuals.*

### RELIGIOUSNESS, PRACTICING RELIGION, AND CHARACTER STRENGTHS

For the comparison of the three groups regarding the character strengths, we conducted a MANOVA with the 24 strengths as dependent variables and the independent factor group (religious and practicing, religious and not practicing, non-religious individuals). The religious individuals, who practiced their religion, scored higher than the other two comparison groups on 16 of the 24 strengths (i.e., curiosity, love of learning, persistence, zest, love, kindness, teamwork, leadership, forgiveness, modesty, prudence, self-regulation, appreciation of beauty, gratitude, hope, and religiousness). The most pronounced differences were found for religiousness (large effect), gratitude (medium effect), love, forgiveness, hope, kindness, and appreciation of beauty (all $\eta_{p}^{2}$*s* > 0.011, see **Tables 1** and **2**). The comparison of non-religious individuals and religiously affiliated individuals, who do not practice their religion, showed no substantial differences regarding the 24 character strengths (all $\eta_{p}^{2}$*s* < 0.01).

### RELIGIOUSNESS, PRACTICING RELIGION, AND ORIENTATIONS TO HAPPINESS

We compared pleasure (P), engagement (E), and meaning (M) of non-religious participants, religiously affiliated participants, who practice their religion and those who did not practice religion. The MANOVA with the three scales of the OTH (P, E, and M) showed that religious individuals, who practice their religion, scored higher on meaning and lower on pleasure than the other two groups, which did not differ from each other (see **Tables 1** and **2**). No substantial differences were found for engagement.

## DISCUSSION

The aim of the present research was to examine how practicing religion relates to variables that designate the “good life”. Therefore, we investigated whether religiously affiliated individuals, who practice vs. those who do not practice their religion and non-religious individuals, differed with regard to their satisfaction with life, their character strengths and their OTH.

The findings of our study show that satisfaction with life was, as predicted, higher in individuals, who reported to practice their religion compared to the other respondents. Moreover, those individuals who are members of a religious community but do not practice their religion, did not differ from non-religious individuals regarding their life satisfaction. Apparently, people do not benefit from being religiousness unless they also engage in practicing their religion actively. The present results fit well with the findings of previous studies on religiousness and well-being that already noted the importance of carrying out religious practices (e.g., Sethi and Seligman, 1993; Mochon et al., 2008). The results of the present study are also in line with positive psychological research supporting the notion of a positive relationship between the frequent usage of one’s strengths, fulfillment and satisfaction with life (e.g., Seligman et al., 2005).

In terms of religiousness and practicing religion, the processes leading to a high degree of well-being in religious people might be multifaceted. It is possible that religiousness has an indirect influence on life satisfaction through people’s dispositional forgiveness. Forgiveness has been shown to correlate with religiousness and satisfaction with life (McCullough et al., 2001; Brown and Phillips, 2005; Jones, 2006). The findings of Brown and Phillips (2005) also indicate that forgiveness is positively related to mental health. Delle Fave et al. (2013) argue that most religious systems recommend healthy lifestyles and cite various studies that provided evidence for the beneficial influence of religiousness (e.g., religious practice) on mental and physical health (see Delle Fave et al., 2013, for an overview). Powell et al. (2003) investigated the relationship between religiousness and mortality. They reviewed several studies and concluded that practicing religion (e.g., church attendance) reduced mortality. Chida et al. (2009) reported similar results. Altogether, practicing religion seems to influence people’s well-being directly as well as indirectly via different mechanisms.

Regarding the relation of religion and character strengths, the findings reported herein showed that individuals, who practiced their religion, scored higher on several character strengths. As expected, religiousness, gratitude, kindness, and forgiveness belonged to the strengths that were mostly pronounced in religious individuals, who indicated that they practice their religion. Other strengths on which these individuals scored higher, were love, hope, and appreciation of beauty. Small effects were also found in association with curiosity, love of learning, persistence, zest, teamwork, leadership, modesty, prudence, and self-regulation. According to these results, engaging in religious practices seems to be beneficial in the development of several strengths. As described earlier, the compilation of the 24 character strengths was based on religious literature to a great extent. Following this, the results showing that 16 of the 24 strengths were increased in individuals carrying out religious practices are not that surprising. A closer inspection reveals that some of the effects were quite small. However, for four of the 24 strengths, we expected stronger effects, an expectation that was confirmed by our data. These strengths are religiousness, gratitude, kindness, and forgiveness. Our results are in line with previous results that reported positive relations between religiousness and gratitude, as well as forgiveness (Jones, 2006; Lambert et al., 2009).

The present findings also fit well with previous research on religiousness and personality. In the recent past, positive correlations were found, for instance, for *agreeableness* (one of the Big Five traits) and the character strengths *kindness* and *forgiveness* (Macdonald et al., 2008). These correlations are quite reasonable, since agreeableness reflects a general prosocial orientation including qualities such as altruism, kindness, and trust. Also, a meta-analysis showed positive relations between the religiousness-measures and *agreeableness* (Saroglou, 2010). Thus, given that agreeableness is related to the strengths kindness and forgiveness as well as to religiousness measures, it is not surprising that we found an increased kindness and forgiveness in religious people, who practice their religion.

We know from previous research that the Big Five trait, agreeableness, is also positively related to life satisfaction (DeNeve and Cooper (1998). Thus, one might wonder whether agreeableness could account for the relation between religiousness and satisfaction with life. The findings of Ciarrocchi et al. (2008), however, contradict this conclusion. They found that religious practices, relational faith and other factors of religiousness scales were significant predictors of hope and optimism above and beyond the Big Five variables.

Another purpose of the present research was to examine the OTH of religiously affiliated individuals that practice vs. those who do not practice their religion – and non-religious individuals. The findings showed that pleasure was lower, whereas meaning was higher in individuals engaging in religious practices compared to those, which did not practice their religion and the non-religious participants. These findings support the findings of previous studies that reported a negative relationship between religiousness and pleasure and a positive relationship between religiousness and meaning (Peterson et al., 2007). Our study extends previous research, because we could show that a religious affiliation alone does not make a difference regarding the life of pleasure and meaning. As predicted, non-religious individuals and those who were religiously affiliated without practicing their religion did not differ from each other. Put differently, only people who practice their religion, seem to have an advantage regarding their feeling for the meaning of life – and thus regarding their happiness.

### LIMITATIONS OF THE PRESENT RESEARCH

Our assessment of practicing religion was oversimplified (i.e., yes, no, no religion). Therefore, we do not know exactly what kind of religious practices the respondents undertook in their lives. Some might have thought of church attendance, others might have considered that personal prayer or the reading of religious literature is a way of practicing one’s religion. Since we do not know what specific religious practices our participants carried out, it is not possible to analyze, whether or not there were differences regarding religious practices in terms of satisfaction with life. Some religious practices might be more important than others with respect to peoples’ life satisfaction or their character strengths. Hence, future research should use a more refined assessment of the various religious practices.

Another limitation of our study is that it is impossible to infer the direction of causality of the effects. All variables were assessed only once and the study did not include any manipulation of the independent variables. However, since other studies using a longitudinal design have already shown that religiousness has a causal influence on life satisfaction (Headey et al., 2010), we assume that practicing religion has a causal effect on life satisfaction and the character strengths as well. Future studies addressing these phenomena are clearly warranted.

### CONCLUSION AND PRACTICAL IMPLICATIONS OF THE PRESENT RESEARCH

The results of the present study indicate that practicing (as opposed to merely passively “belonging to” a) religion does indeed make a difference regarding an individuals’ degree of satisfaction with life, their character strengths and their evaluation of the meaning in life. Drawing the distinction between people who actively practice their religion vs. those who consider themselves to be part of a religion but do not actively practice it enabled us to examine the importance of practicing one’s religion. The findings of the present research provide evidence for the widely shared assumption that the cultivation of one’s strengths is beneficial. Aside to being more satisfied with one’s life, it is possible that some of the other corresponding character strengths might be fostered as well.

Up to the present time, we do not know if it is possible that practicing a particular strength can backfire under some specific circumstances. Even though the present findings clearly indicate a positive influence of practicing religion on life satisfaction, adverse effects might also be possible. Imagine someone who, for example, is forcing herself/himself to attend church every week, although that exercise does not really mean anything to her/him. The common expectation would, of course, be that the person would cease that behavior. The possibility, nonetheless, exists that some people might cultivate behaviors corresponding to character strengths that do not match their personal characters and thus, might decrease their feelings of well-being. Testing this hypothesis remains a challenge for future research.

Finally, it is important to note that our respondents were from countries (i.e., Germany, Switzerland, and Austria) that are characterized by a declining development regarding the organized religions. Thus, it would be interesting to conduct another study after a certain period of time. If the declining development continues, the percentage of people who do not actively practice their religion should diminish. As they do not benefit from being religiously affiliated, they are least likely to continue.

## Conflict of Interest Statement

The authors declare that the research was conducted in the absence of any commercial or financial relationships that could be construed as a potential conflict of interest.
